# Metabolic syndrome status over 2 years predicts incident chronic kidney disease in mid-life adults: a 10-year prospective cohort study

**DOI:** 10.1038/s41598-018-29958-7

**Published:** 2018-08-16

**Authors:** So Jin Lee, Hun Ju Lee, Hyun jeong Oh, Taehwa Go, Dae Ryong Kang, Jang Young Kim, Ji Hye Huh

**Affiliations:** 1 Yonsei University, Wonju College of Medicine, Wonju, Korea; 2 Center of Biomedical Data Science, Yonsei University, Wonju College of Medicine, Wonju, Korea; 3 Department of Internal Medicine, Yonsei University, Wonju College of Medicine, Wonju, Korea

## Abstract

We investigated whether changes in MetS status over two years modify the 10-year risk of CKD and proteinuria. A prospective cohort study was conducted in 7,251 subjects without CKD at baseline. We categorized subjects according to MetS status over two years: non-MetS (no MetS at either visit), intermittent MetS (positive for MetS at one assessment), and persistent MetS (positive for MetS at two assessments). The hazard ratio (HR) of new-onset CKD over 10-year was calculated using Cox models. During the 10-year follow-up period, 923 (12.7%) developed CKD. Compared to the non-MetS group, the fully adjusted HR for new-onset CKD was the highest in the persistent MetS group (HR, 1.53; 95% CI, 1.23–1.90), followed by the intermittent MetS group (HR, 1.29; 95% CI, 1.04–1.59) (P for trend <0.001). The HR for developing proteinuria was 1.79 (95% CI, 1.15–2.79) in the persistent MetS group and 0.70 (95% CI, 0.42–1.19) in the intermittent MetS group when the non-MetS group was considered as the reference group. Temporal changes in MetS status over two years influenced the 10-year risk of incident CKD and proteinuria. Our findings suggest that monitoring and strictly controlling MetS are important in preventing renal function decline.

## Introduction

Chronic kidney disease (CKD) is a major risk factor for end-stage renal disease (ESRD), cardiovascular disease, and premature death^1–5^. According to data from the Third National Health and Nutrition Examination Survey (NHANES III), there are approximately 8.3 million (4.6%) adult patients with CKD in America^1^, and the prevalence of CKD has been estimated as 7.9% in Korea^6^. Consequently, CKD has become an important public health problem worldwide. The rapidly increasing prevalence of CKD cannot be fully explained by the already-known single risk factors such as diabetes and hypertension, suggesting that there are other contributing variables^7^. Therefore, identifying modifiable predictors may help to decrease the prevalence of CKD along with its economic and social burden.

Recent prospective studies and meta- analyses have shown that the presence of metabolic syndrome (MetS) at baseline is a risk factor for developing CKD^8^. However, MetS status in individuals may change over time through factors such as lifestyle modification. Indeed, previous large-scale studies showed that there were considerable changes in the MetS status of individuals over follow-up periods^9–11^. Ohnishi *et al*. reported that long-term presence of MetS status, rather than MetS status at a given moment, is more closely associated with a higher risk of diabetes^10^. From these findings, we hypothesized that the change patterns of MetS status over time may vary among individuals; thus, longitudinal change of MetS may have independent impact on the renal dysfunction. However, the relationship of longitudinal changes in MetS with renal function decline has not been investigated in a large scale epidemiologic study. Furthermore, the relationship of MetS status duration with incident proteinuria, a clinical indicator of kidney function decline before CKD onset^12,13^, has not been determined until now.

Therefore, the aim of this study was to determine the independent association of the change of MetS status, based on two assessments of MetS status over 2 years, with the 10-year risk of CKD in a large cohort study of an apparently healthy population.

## Results

### Baseline characteristics

Table 1 shows the baseline biochemical and clinical characteristics, categorized by the MetS status change groups. The total number of participants was 7,251, of whom 4,416 (60.9%) were in the non-MetS group, 1,431 (19.7%) were in the intermittent MetS group, and 1,404 (19.4%) were in the persistent MetS group. The age, blood pressure, body mass index, and waist circumference and the levels of triglyceride, fasting glucose, and HbA1c were highest in the subjects with persistent MetS, followed by intermittent MetS and then non-MetS (P < 0.001). Conversely, HDL-C levels were highest in the non-MetS group, followed by intermittent MetS and then persistent MetS (P < 0.001). Total cholesterol levels, eGFR, and hsCRP were significantly lower in the non-MetS group than in the other two groups. LDL cholesterol levels and the frequency of regular exercise were similar among the three groups. Daily alcohol intake amount was higher in intermittent MetS group, followed by non-MetS group and persistent MetS group, in that order (P = 0.045). Current smokers were significantly lower in the persistent MetS group than the other groups.

**Table 1:** Baseline characteristics of study participants according to changes in metabolic syndrome status over 2 years.

	Non-metabolic syndrome (N = 4416)	Intermittent metabolic syndrome (N = 1431)	Persistent metabolic syndrome (N = 1404)	P-value
Age (years)	50.4 ± 8.4^†‡^	53.5 ± 8.6^*‡^	55.4 ± 8.4^*†^	<0.001
Sex, male (%)	2262 (51.2)	675 (47.2)	527 (37.5)	<0.001
SBP (mm Hg)	118.4 ± 16.1^†‡^	129.3 ± 17.6^*‡^	136.9 ± 17.6^*†^	<0.001
DBP (mm Hg)	79.2 ± 10.6^†‡^	85.7 ± 11.0^*‡^	89.8 ± 10.7^*†^	<0.001
BMI (kg/m2)	23.6 ± 2.7^†‡^	25.6 ± 2.8^*‡^	27.0 ± 2.8^*†^	<0.001
Waist circumference (cm)	79.0 ± 7.5^†‡^	86.1 ± 7.1^*‡^	90.9 ± 7.2^*†^	<0.001
Total cholesterol (mg/dL)	187.1 ± 33.8^†‡^	194.2 ± 35.5*	197.3 ± 34.3^*^	<0.001
TG (mg/dL)	128.5 ± 71.3^†‡^	184.7 100.4^*‡^	231.4 130.8^*†^	<0.001
HDL-C (mg/dL)	47.5 ± 10.0^†‡^	42.0 ± 8.5^*‡^	38.4 ± 6.9^*†^	<0.001
LDL-C (mg/dL)	113.9 ± 31.2	115.3 ± 34.4	112.7 ± 35.0	0.099
Fasting glucose (mg/dL)	83.4 ± 14.1^†‡^	89.8 ± 25.3	93.4 ± 24.4^*†^	<0.001
HbA1c (%)	5.6 ± 0.6^†‡^	5.9 ± 0.9^*‡^	6.2 ± 1.1^*†^	<0.001
eGFR	94.0 ± 13.1^†‡^	91.8 ± 12.5*	90.9 ± 12.5^*^	<0.001
hsCRP (mg/L)	2.0 ± 4.2^†‡^	2.4 ± 4.0*	2.8 ± 7.2^*^	<0.001
Regular exercise (%)	648 (14.7)	211 (14.7)	179 (12.8)	0.175
Alcohol intake (g/day)	9.4 ± 20.2	10.0 ± 21.7	8.1 ± 20.9	0.045
Smoking status (%)				
Current smoker	1141 (26.1)^&^	352 (25.0)^&^	283 (20.4)^#$^	<0.001

Data are presented as mean ± standard deviation or n (%) for categorical variables.

MetS, metabolic syndrome; SBP, systolic blood pressure; DBP, diastolic blood pressure; BMI, body mass index; HDL, high-density lipoprotein; LDL, low-density lipoprotein; HbA1c, glycated hemoglobin; BUN, blood urea nitrogen; hsCRP, high-sensitivity c-reactive protein.

^*^P < 0.05 vs. Non-MetS after ANOVA followed by Scheffé post hoc comparison.

^†^P < 0.05 vs. Intermittent MetS after ANOVA followed by Scheffé post hoc comparison.

^‡^P < 0.05 vs. Persistent MetS after ANOVA followed by Scheffé post hoc comparison.

^#^P < 0.05 vs. Non-MetS after Bonferroni correction.

^$^P < 0.05 vs. Intermittent MetS after Bonferroni correction.

^&^P < 0.05 vs. Persistent MetS after Bonferroni correction.

### The risks of incident CKD according to the change in metabolic syndrome status over 2 years

Table 2 shows the Cox regression results for incident CKD categorized by the MetS status change groups. Among 7,251 subjects, CKD developed in 923 (12.7%) during the 10-year follow-up period. When the non-MetS group was considered as the reference group, the crude HR of the intermittent MetS group was 1.97 (95% CI, 1.67–2.32) and that of the persistent MetS group was 2.83 (95% CI, 2.43–3.28) (*P* for trend <0.001). The HR of the intermittent MetS group was 1.49 (95% CI, 1.26–1.78) and that of the persistent MetS group was 1.79 (95% CI, 1.52–2.10) after adjustment for age (continuous variable), sex (male/female), smoking (current/former/never), alcohol intake (continuous variable), and regular exercise (yes/no). After further adjustment for body mass index (continuous variable), hsCRP (log-transformed continuous variable), eGFR (continuous variable) and diabetes (yes/no) at baseline, the HRs were 1.29 (95% CI, 1.04–1.59) for the intermittent MetS group and 1.53 (95% CI, 1.23–1.9) for the persistent MetS group (model 2). These findings indicated that the subjects with persistent MetS over 2 years experienced the highest risk of developing CKD among all three MetS status change patterns.

**Table 2:** Association between 2-year metabolic syndrome status and incidence of chronic kidney disease.

	Non- metabolic syndrome	Intermittent metabolic syndrome	Persistent metabolic syndrome	P for trend
Incident CKD case	382 (8.7)	229 (16.0)	312 (22.2)	<0.001
Crude hazard ratio	Reference	1.97 (1.67–2.32)	2.83 (2.43–3.28)	<0.001
Model 1	Reference	1.49 (1.26–1.78)	1.79 (1.52–2.10)	<0.001
Model 2	Reference	1.29 (1.04–1.59)	1.53 (1.23–1.90)	<0.001

Model 1: adjusted for age, sex, smoking, alcohol intake and regular exercise.

Model 2: Model 1+ BMI, hsCRP, eGFR and diabetes.

CKD, chronic kidney disease; BMI, body mass index; hsCRP, high-sensitivity c-reactive protein; eGFR, estimated glomerular filtration rate.

### Kaplan–Meier plot of time to disease according to the two-year changes in MetS status

**Figure 1:**
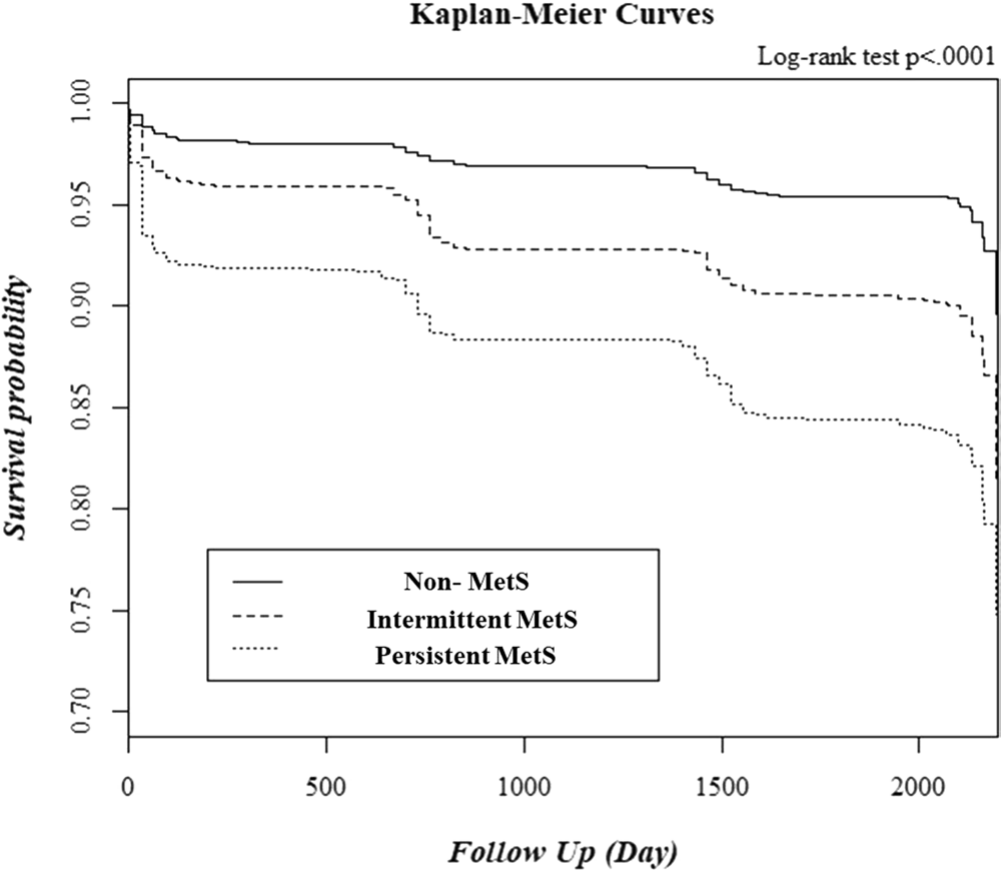
Chronic kidney disease-free survival duration according to change in metabolic syndrome status from baseline to 2 years by Kaplan–Meier analysis. MetS, metabolic syndrome.

The Kaplan–Meier survival curves for cumulative CKD-free survival during the 10-year follow-up period for the MetS status groups are shown in Fig. 1. Compared with the non-MetS group, the intermittent MetS and persistent MetS groups had higher probabilities of developing incident CKD. The cumulative incidence of CKD significantly increased to the greatest degree for the persistent MetS group, followed by the intermittent MetS group and then the non-MetS group (log-rank test, P < 0.001 for all three comparisons). The survival curves were significantly different between the intermittent MetS and persistent MetS groups (log-rank test, P < 0.001). This result indicates that subjects with intermittent MetS had a significantly lower risk of incident CKD than the subjects with persistent MetS during follow-up period.

### Risk of incident proteinuria according to the changes in MetS status

Using the Cox proportional hazards model, we also investigated the independent risk of change of MetS status, based on repeated longitudinal assessment of MetS status over 2 years, for the development of proteinuria (Table 3). During the 10-year follow up period, incident proteinuria developed in 209 (2.8%) of the 7,251 participants. When the non-MetS group was used as a reference, persistent MetS status was associated with incident proteinuria, even after the adjustment for multiple confounders, which yielded an adjusted HR of 1.79 (95% CI, 1.15–2.79). Risk of incident proteinuria was attenuated with insignificant adjusted HR of 0.70 (95% CI, 0.42–1.19) in the intermittent MetS group (model 2).

**Table 3:** Association between two-year metabolic syndrome status and incidence of proteinuria.

	Non- metabolic syndrome	Intermittent metabolic syndrome	Persistent metabolic syndrome	P for trend
Incident proteinuria case	107 (2.4)	34 (2.4)	68 (4.9)	<0.001
Crude hazard ratio	Reference	1.07 (0.73–1.58)	2.26 (1.67–3.07)	<0.001
Model 1	Reference	0.91 (0.59–1.40)	2.32 (1.68–3.20)	<0.001
Model 2	Reference	0.70 (0.42–1.19)	1.79 (1.15–2.79)	<0.001

Model 1: adjusted for age, sex, smoking, alcohol intake and regular exercise.

Model 2: Model 1+ BMI, hsCRP, eGFR and diabetes.

BMI, body mass index; hsCRP, high-sensitivity c-reactive protein; eGFR, estimated glomerular filtration rate.

## Discussion

In this large prospective cohort study of Korean adults, we observed the relationship of MetS status changes over 2 years with renal function decline during a 10-year follow-up period. Subjects with persistent MetS over 2 years had the greatest risk of developing CKD and proteinuria during that period. At the same time, subjects with intermittent MetS (resolution of MetS or incident MetS) had a relatively attenuated risk of incident CKD and proteinuria compared with persistent MetS. To the best of our knowledge, this is the largest community-based longitudinal cohort study to report the relationship of late changes in MetS over a short-term period with the further occurrence of CKD. Although MetS has been known for decades to be a risk factor for CKD, these findings suggest that an individual’s early patterns of change in MetS status may provide additional information about his or her risk of development of CKD.

30% of Korean adults have MetS and its prevalence is more increasing^14^. From this background, previous reports have demonstrated the clinical implication of MetS in the future development of CKD^15–17^. However, these studies only investigated the association between MetS based on single assessments at baseline and indent CKD. In addition, most of the previous studies which examined the impact of changes in MetS on individual’s health had relatively short follow-up periods (shorter than 2 years) and these are small-scale studies. For example, a recent randomized control study showed that a six-month lifestyle change program reduced the number of MetS components with decreasing measures of insulin resistance and systemic inflammation^18^. Similarly, our large-scale cohort study also found that a considerable number of subjects experienced changes in their MetS status during a 2-year follow-up period (19.7%). This result provides a unique insight into longitudinal change patterns of MetS status, and highlights that MetS status changes are heterogeneous over time within the Korean adult population. Furthermore, Lin *et al*. reported that changes in MetS status during 24 month associated with rapid renal function decline in CKD patients during same period from retrospective longitudinal data^19^. However, to date, the relationship of renal dysfunction with short-term changes in MetS status in the general population is unknown.

From this background, our study investigated the independent association of the change of MetS status with the development of CKD, based on two repeated longitudinal assessments of MetS status over 2 years. We observed that intermittent MetS status over 2 years is associated with an increased risk of developing CKD over 10 years compared to non-MetS status. This finding is in line with those of previous studies^15–17^. Our findings confirm and extend the implication of MetS as an important risk factor for the pathogenesis of renal function deterioration. However, unlike previous studies, our study additionally demonstrated the increased risk of CKD with intermittent MetS was attenuated compared to the risk with persistent MetS during the same time.We also observed the risk of developing proteinuria, well established indicator of CKD and potentially modifiable risk factor for CKD^20^, was not higher in the intermittent MetS group compared to the non-MetS group. It should be noted that even after adjustment for confounding factors, the risk for developing CKD and proteinuria of intermittent MetS remained attenuated. These findings suggest that regularly monitoring MetS status among individuals is of significance for public health and clinical practice because these individuals are generally not treated although they may be at a high risk of developing CKD later. This is particularly important, given that lifestyle intervention could effectively resolve MetS status.

Besides the well-known mechanisms (i.e., systemic inflammation, oxidative stress, and insulin resistance)^21–24^, several other mechanisms may explain the association between changes in MetS status over a short-term period and long-term risk of CKD. First, this phenomenon can be partly explained by the concept of “cardiometabolic memory”^25^. For example, transient intensive lowering of glucose appeared to induce memory for the suppression of diabetic microangiopathies and memory for the suppression of microangiopathies in post-trial follow-up periods^26,27^. Also, transient intensive blood pressure and cholesterol lowering were associated with the formation of memory for the suppression of cardiovascular events^28–30^. Potential mechanisms for propagating this “cardiometabolic memory” comprise the non-enzymatic glycation of cellular proteins and lipids, in particular mitochondrial proteins, in conjunction with excess cellular reactive oxygen and nitrogen species that act in concert to maintain stress signaling^31^. Consequently, “cardiometabolic memory” could be a consequence of structural and functional changes that, while occurring in the early metabolic environment, also carry implications regarding the later development of CKD^32^. This concept provides further support for initiating proper interventions for subjects with metabolic abnormalities as early as possible for the prevention of CKD. Following this strategy, not only immediate and short-term but long-term beneficial effects could be expected.

Although we included a large number of participants and used long-term follow-up data, this study had several limitations. First, GFR was not directly measured but was estimated using a serum creatinine-based equation, which might have overestimated or underestimated the actual GFR^33^. Second, we could not categorize the intermittent MetS group more specifically into a resolved MetS group and an incident MetS group, due to the low number of incident CKD cases in each group. Third, we defined proteinuria as having a result of more than 1+ using a dipstick: that result may have been due to other physiological reasons. Thus, a random measurement error in assessment of proteinuria may have occurred depending on the timing of the measurement, which may have influenced our results^34,35^. However, as we excluded all subjects with urinary protein ≥+1 on dipstick urinalysis at baseline, we only included subjects who had a very low risk of CKD. Therefore, we could demonstrate more definitively the independent association between MetS and CKD. Fourth, we did not collect complete information regarding the types of anti-hypertensive drugs and the usage of medications that may have affected renal function or serum creatinine. “Fifth, because of the nature of cohort data, we cannot detect the exact time at incidence of CKD and it makes a time bias in survival analysis”.

Finally, because the current study included only Korean adults living in a specific community, our result may not be generalizable to other populations. However, the homogeneous nature of our cohort could help to reduce potential confounding due to racial and health care disparities and, therefore, enhance internal validity, which is a prerequisite for generalizability.

Collectively, this study demonstrated that changes in MetS status over 2 years were significantly associated with subsequent risk of developing CKD and proteinuria in a 10-year prospective study. Subjects with intermittent MetS had a lower risk of renal function deterioration compared with subjects with persistent MetS. This finding indicates the importance of improving metabolic profiles through lifestyle changes for people with MetS. Our results also suggest that monitoring MetS status changes may be an important approach to identifying populations at higher risk of renal dysfunction and may help to prevent CKD in clinical practice. Considering that the close relationship between MetS and CKD has recently attracted considerable scientific interest, further studies with different racial and ethnic populations are also warranted to replicate our findings.

## Methods

### Study population and design

**Figure 2:**
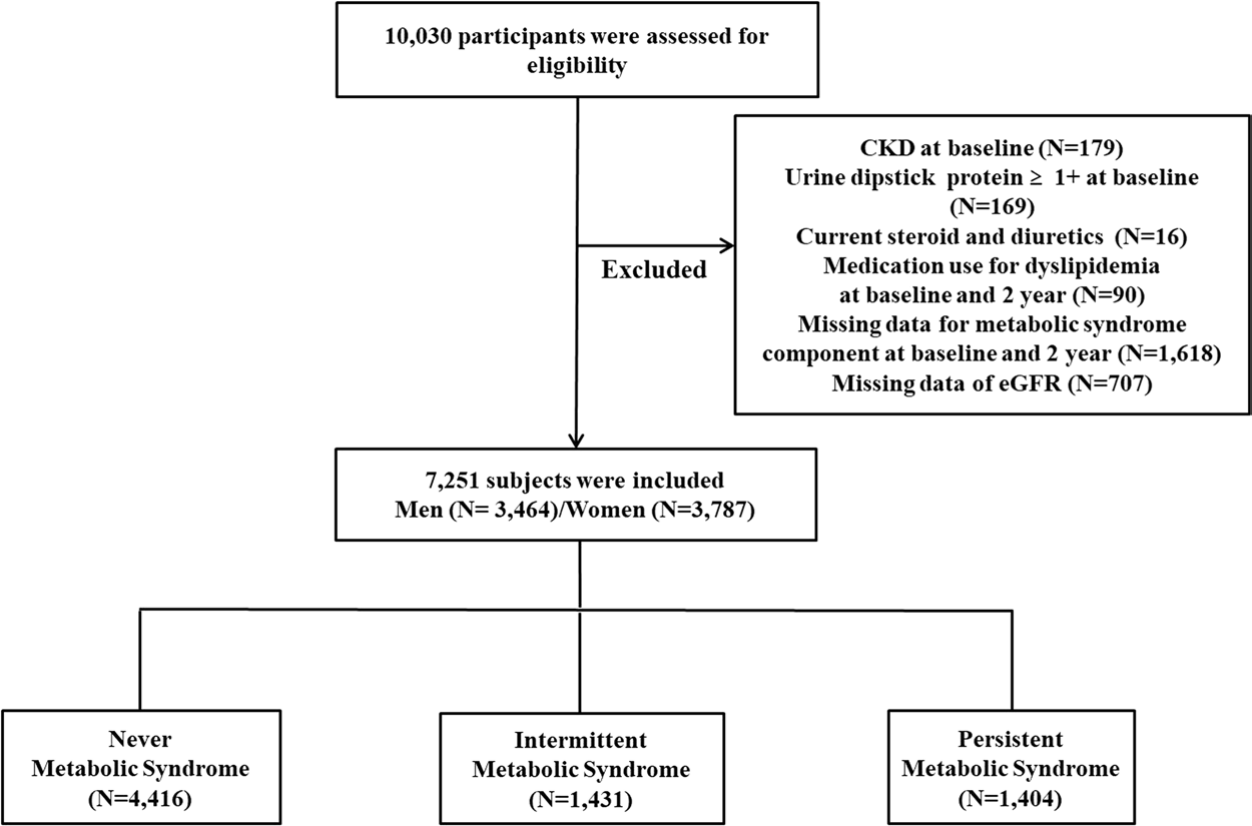
Flow chart. MetS, metabolic syndrome; CKD, chronic kidney disease; eGFR, estimated glomerular filtration rate.

Data for the present study were derived from the Korean Genome and Epidemiology Study (KoGES)-Ansung and Ansan cohort study, a rural community-based prospective study. The Ansung-Ansan study began in 2001 and includes biennial follow-up examinations. A total of 10,030 participants aged 40–69 years were enrolled at baseline^36,37^. Among them, we included subjects who participated in this survey from baseline (2001–2002) to the fifth follow-up visit (2011–2012). We excluded subjects with unknown MetS status in the first and second phase of the survey (N = 1,618). Participants were excluded if they had CKD as defined by estimated glomerular filtration rate (eGFR) < 60 mL/min per 1.73 m^2^ at a baseline health examination (N = 179), had urinary protein ≥+1 on dipstick urinalysis (N = 169), were taking glucocorticoid or diuretics (N = 16), and/or were taking any medication agents for dyslipidemia (N = 90). In addition, participants were excluded if they had missing data on eGFR (N = 707). After exclusion, 7,251 participants were eligible for this study (Fig. 2). At each visit, informed written consent was obtained from all participants. The study protocol was approved by the Ethics Committee of the Korean Center for Disease Control and the Institutional Review Boards of the Yonsei University Wonju College of Medicine (YWMR-15-9-075).

### Data collection

KoGES participants are examined biannually. At those times, they are asked to complete questionnaires on their personal and family medical histories, smoking habits, alcohol consumption, exercise status, and use of medication. For the present study, participants were categorized according to smoking status (never, past, or current smoker) and exercise status (none, irregular exercise defined as ≤3 times per week and regular exercise defined ≥4 times per week). Height, body weight, and waist and hip circumference were measured using standard methods. Waist circumference was measured at the narrowest point between the upper iliac crest and lowest rib after normal expiration. Blood pressure was measured by averaging three measurements taken in the morning after at least 10 min of rest in a sitting position. Diabetes was defined as a fasting glucose concentration of ≥126 mg/dL, a post-load glucose concentration of ≥200 mg/dL after a 75-g oral glucose tolerance test, or taking anti-diabetic medication, based on the American diabetes association criteria^38^.

Laboratory samples were obtained after a 12-h fast. Plasma total cholesterol, triglycerides, high-density lipoprotein (HDL) cholesterol, creatinine, and alanine and aspartate aminotransferases (ALT and AST, respectively) were measured using a Hitachi 747 chemistry analyzer (Hitachi Ltd., Tokyo, Japan). Low-density lipoprotein (LDL) cholesterol was assessed by the Friedewald equation. Levels of high-sensitivity C-reactive protein (hsCRP) were measured using an immunoradiometric assay (ADVIA 1650 analyzer, Bayer Diagnostics, Tarrytown, NY, USA). Serum creatinine was measured using Jaffe’s method with a Hitachi Automatic Analyzer 7600 (Hitachi, Tokyo, Japan).

### Definition of metabolic syndrome and metabolic syndrome status change

The definition of MetS in this study was based on a modification of the National Cholesterol Education Program Adult Treatment Panel III (NCEP-ATP III) criteria [10]. MetS was defined as the presence of three or more of the following components: (1) abdominal obesity, defined as a waist circumference ≥90 cm for males or ≥85 cm for females (following cutoffs for abdominal obesity defined by the Korean Society of Obesity)^39^; (2) hypertriglyceridemia, defined as a serum triglyceride concentration ≥150 mg/dL; (3) low HDL cholesterol, defined as a serum HDL cholesterol concentration <40 mg/dL for males or <50 mg/dL for females; (4) high blood pressure, defined as systolic blood pressure (SBP) ≥ 130 mmHg, diastolic blood pressure (DBP) ≥ 85 mmHg, or treatment with antihypertensive agents; and (5) high fasting glucose, defined as a fasting serum glucose ≥100 mg/dL. MetS status change was assessed as the presence or absence of MetS over 2 years, corresponding to the period baseline and the two-year follow-up visit. According to the changes in MetS status between baseline and the two-year follow-up visit, participants were divided into three groups as follows; a non-MetS to non-MetS group (non-MetS group), a non-MetS to MetS group or a MetS to non-MetS group (intermittent MetS group), and a MetS to MetS group (persistent MetS group).

### Definition of incident CKD and proteinuria

This study calculated eGFR using the CKD-Epidemiology Collaboration (CKD-EPI) equation: eGFR (mL/min per 1.73 m^2^) = 141 × min (creatinine/κ, 1)^α^ × max (creatinine/κ, 1)−1.209 × 0.993^age^ (years) × 1.018 [if female] × 1.159 [if black]: where κ = 0.7 for females and 0.9 for males, α = −0.329 for females and −0.411 for males, min indicates the minimum of creatinine/κ or 1, and max indicates the maximum of creatinine/κ or 1. This study defined incident CKD as occurring if eGFR decreased to less than 60 and decreased by more than 25% at the same time. In addition, incident proteinuria was defined as urine protein ≥1+ on dipstick urinalysis with a baseline of negative or trace.

### Statistical analyses

Continuous variables are presented as mean ± standard deviation, and categorical variables are expressed as numbers (n) and percentages (%). Differences among groups were analyzed using analysis of variance (ANOVA) with Scheffe’s post hoc analysis method for continuous variables and chi-square tests for categorical variables. Cumulative CKD incidence rates were estimated using Kaplan-Meier survival curves, and equality was compared using log-rank tests. Cox proportional-hazards analyses were conducted to estimate the hazard ratio (HR) and 95% confidence interval (CI) for the association between changes in MetS over two years and the development of CKD or proteinuria. A *P* value of <0.05 was considered statistically significant, and SAS 9.2 version (SAS Inc., Cary, NC, USA) was used.
